# Beyond the γ-aminobutyric acid hypothesis of schizophrenia

**DOI:** 10.3389/fncel.2023.1161608

**Published:** 2023-04-24

**Authors:** Kazuyuki Fujihara

**Affiliations:** ^1^Department of Psychiatry and Neuroscience, Gunma University Graduate School of Medicine, Maebashi, Japan; ^2^Department of Genetic and Behavioral Neuroscience, Gunma University Graduate School of Medicine, Maebashi, Japan

**Keywords:** inhibitory neurons, psychiatric disorders, knockout rats, genome editing, microglia

## Abstract

Abnormalities in the γ-aminobutyric acid (GABA) system have been reported in the postmortem brains of individuals with schizophrenia. In particular, the reduction of one of the GABA-synthesizing enzymes, the 67-kDa isoform of glutamate decarboxylase (GAD67), has garnered interest among researchers because of its role in the formation of γ-oscillations and its potential involvement in the cognitive dysfunction observed in schizophrenia. Although several animal models have been generated to simulate the alterations observed in postmortem brain studies, they exhibit inconsistent behavioral phenotypes, leading to conflicting views regarding their contributions to the pathogenesis and manifestation of schizophrenia symptoms. For instance, GAD67 knockout rats (also known as *Gad1* knockout rats) exhibit marked impairments in spatial working memory, but other model animals do not. In this review, we summarize the phenotypic attributes of these animal models and contemplate the potential for secondary modifications that may arise from the disruption of the GABAergic nervous system.

## 1. Introduction

Schizophrenia is one of the most common psychiatric disorders with a lifetime morbid risk of approximately 0.72% (Saha et al., 2005). Although there are exceptions, schizophrenia generally develops during adolescence and young adulthood and has a chronic course. The symptoms of schizophrenia are highly varied; however, there exist several characteristic clusters of symptoms (Liddle, 1987). The first cluster is called the positive symptom or reality distortion syndrome. This comprises hallucinations and delusions. The second is negative symptoms or psychomotor poverty syndrome, which includes avolition and poor emotional expression (Nakaya and Ohmori, 2008; Correll and Schooler, 2020). The third is the disorganization syndrome, which represents conceptual disorganization and bizarre behavior. In addition, impairments in cognitive function, such as working memory deficits, are considered important prognostic factors (Keefe and Harvey, 2012). Psychomotor poverty and disorganization syndrome are also associated with cognitive impairment (Altshuler et al., 2004; Liddle, 2019). It is noteworthy that these various symptoms are not unique to schizophrenia but also occur with certain types of encephalitis and substance abuse (e.g., amphetamines and phencyclidine). Therefore, clinicians must rule out organic diseases or drug effects during diagnosis (American Psychiatric Association, 2013). Conversely, this implies that unknown biological factors may be responsible for schizophrenia. Indeed, since Kraepelin’s conceptualization, it has been assumed that there is some biological basis for this disorder (Hoenig, 1983). Therefore, several pathophysiological hypotheses have been proposed.

The classical hypothesis of schizophrenia is the dopamine hypothesis (Howes and Kapur, 2009). This was proposed based on the finding that antipsychotics are dopamine D_2_ receptor antagonists (Meltzer and Stahl, 1976; Seeman, 2021). The dopamine hypothesis is consistent with the fact that amphetamines, which increase extracellular dopamine levels, produce positive symptom-like symptoms. However, the presence of treatment-resistant cases and the limited efficacy of the drug in treating negative symptoms and cognitive dysfunction make it difficult to explain the mechanisms of schizophrenia through the dopaminergic system alone (Correll and Schooler, 2020). Another promising hypothesis is the glutamate hypothesis (Uno and Coyle, 2019), as the N-methyl-D-aspartate (NMDA)-type glutamate receptor antagonist phencyclidine causes both positive and negative symptoms. In addition, encephalitis, in which autoantibodies to the NMDA receptor are produced, causes psychiatric symptoms similar to schizophrenia (Dalmau et al., 2011). Although various other pathological hypotheses have been proposed, a unified understanding has not yet been reached. The concept of schizophrenia is based on its symptoms and course, and there are currently no biomarkers. It is crucial to keep in mind that there is no evidence that schizophrenia is a single biological disease but only a clinical syndrome.

As mentioned above, dopamine and glutamate hypotheses were proposed based on pharmacological findings. In contrast, the γ-aminobutyric acid (GABA) hypothesis is based on results in postmortem brain studies (Gonzalez-Burgos and Lewis, 2008; Lewis and Sweet, 2009; Dienel et al., 2022). GABA, an inhibitory neurotransmitter, is biosynthesized by decarboxylation of glutamate (Bu et al., 1992; Soghomonian and Martin, 1998; Obata, 2013). This reaction is catalyzed by glutamate decarboxylase (GAD), which is encoded by two different genes, *GAD1* and *GAD2* in humans (*Gad1* and *Gad2* in rodents). These genes correspond to the 67-kDa isoform (GAD67) and the 65-kDa isoform (GAD65), respectively. Of the two isoforms, GAD67 has been reported to be decreased in the postmortem brains of individuals with schizophrenia. In addition, other marker molecules associated with GABAergic neurons are also altered in expression. The replicability of GABAergic nervous system disturbances observed in the cerebral cortex and hippocampus has attracted the attention of many researchers over the past two decades. In particular, GABAergic systems are involved in working memory through the formation of γ-oscillations, which have been postulated to be associated with cognitive dysfunction in schizophrenia (Gonzalez-Burgos et al., 2010).

In recent years, the pathologies in the neurotransmitters and neurons as well as glial cells, including microglia (Volk, 2017; Murphy et al., 2021; Parellada and Gassó, 2021; Dienel et al., 2022) and astrocyte (Notter, 2021; de Oliveira Figueiredo et al., 2022), have also been implicated in schizophrenia. For example, the activation of microglia has been reported in schizophrenia (Radewicz et al., 2000; Wierzba-Bobrowicz et al., 2005; Fillman et al., 2013; Bloomfield et al., 2016; Marques et al., 2019). Interestingly, some microglia express GABA receptors and can monitor ambient GABA concentrations (Kuhn et al., 2004). Therefore, some researchers refer to GABA as a “neurotransmitter” that mediates signaling between neurons and microglia (Barragan et al., 2015). Impairment of GABAergic neurotransmission in schizophrenia can affect glial cells.

With these considerations in mind, this mini-review first outlines the GABA hypothesis and the animal models based on this hypothesis. This review will primarily focus on GAD67 because it directly affects GABA synthesis, has been independently reported to be downregulated in different postmortem brain banks, and various types of genetically modified animal models have been generated. Further, we discuss the potential of the neuroimmune system as a critical player that interacts with the GABAergic nervous system to further the pathogenesis of the disorder and discuss directions for future research.

## 2. GABA hypothesis of schizophrenia

**FIGURE 1:**
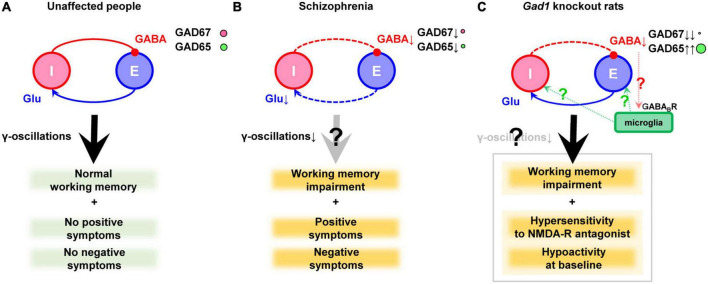
GABAergic dysfunction in the cortex of schizophrenia and *Gad1* knockout rats. **(A)** Balanced neurotransmission of excitatory (E) glutamate (Glu) and inhibitory (I) GABA. **(B)** Impaired inhibitory transmission caused by GAD67 (plus GAD65) reduction in schizophrenia. Deficient excitatory input probably exists as well. Notably, the cause and result relationship between the impaired transmission and clinical symptoms cannot be demonstrated only postmortem studies alone. **(C)** Deficient inhibitory transmission caused by complete loss of GAD67 in *Gad1* knockout rats. The compensatory increase in GAD65 cannot prevent behavioral abnormalities relevant to schizophrenia. The presence or absence of γ-oscillations abnormalities has not yet been verified. In addition, a hypothetical involvement of microglia is also shown (see the main text). Causal relationships that have not been directly proven are indicated with question marks to denote their hypothetical nature.

In the 1970s, a reduction in GAD activity and a decrease in GABA content in the nucleus accumbens and thalamus were reported, which marked the inception of the GABA hypothesis (Bird et al., 1977; Perry et al., 1979). Later, brain banks were established, and the mRNA and protein levels of genes related to the GABAergic nervous system were more precisely analyzed. In the prefrontal cortex of schizophrenia, many studies have documented a reduction in GAD67 mRNA (Akbarian et al., 1995; Guidotti et al., 2000; Volk et al., 2000; Hashimoto et al., 2003, 2008b,a; Veldic et al., 2005; Ruzicka et al., 2007; Tsubomoto et al., 2019) or protein levels (Guidotti et al., 2000; Curley et al., 2011, 2013; Fish et al., 2021). The expression of GAD67 has also been reported to be decreased in the temporal cortex (Impagnatiello et al., 1998), the hippocampus (Heckers et al., 2002), and the cingulate cortex (Woo et al., 2007, 2012). Although it has received little attention, a recent report also suggested decreased level of GAD65 in GABAergic terminals (Fish et al., 2021). However, GAD65 is outside the scope of this review. It has also been reported that the impairment of GABAergic neurons is accompanied by decreased input from excitatory neurons (Chung et al., 2016a,b). These findings are illustrated in Figures 1A, B.

Decreased GAD67 expression is particularly prominent in parvalbumin-positive cells (PV neurons) among the inhibitory interneurons (Hashimoto et al., 2003; Curley et al., 2011). PV neurons are major GABAergic neurons, accounting for approximately 25% of all GABAergic neurons in humans and 40% in rodents (Tamamaki et al., 2003; Uematsu et al., 2008). Since PV neurons (especially PV basket cells) play an essential role in synchronizing action potentials within the neuronal population during working memory tasks (γ-oscillations; Gonzalez-Burgos and Lewis, 2008; Gonzalez-Burgos et al., 2010), GAD67 reduction is hypothesized to be the origin of cognitive impairment in schizophrenia. In addition to GAD67, PV expression is reduced in the postmortem brains of patients with schizophrenia (Hashimoto et al., 2003). Other findings include decreased expression of somatostatin, another marker of GABAergic neuron subtypes, reduced expression of the GABA-A receptor α1 subunit, the δ subunit, GABA transporter (GAT-1), neuropeptide Y, and cholecystokinin (Hashimoto et al., 2008a,b). The reduced expression of GAT-1 has been suggested to occur at PV neuron terminals (Bitanihirwe and Woo, 2014), further supporting the involvement of PV neurons in the pathogenesis of schizophrenia.

Moreover, attempts have been made to measure GABA concentrations in the brains of people with schizophrenia *in vivo*. Nuclear magnetic resonance spectroscopy (MRS) has been used to detect and quantify substances in the human brain. In standard clinical MRS, traces of GABA are buried in the signals of other substances, but a particular sequence called MEGA-PRESS makes it possible to measure it (Rothman et al., 1993). Our group has also used this technique to reveal correlations between impulse control and GABA concentrations in healthy subjects and discussed their relationship with a predisposition to psychiatric disorders (Fujihara et al., 2015b). Several GABA measurements have also been made in schizophrenia using GABA-MRS (Yoon et al., 2010, 2020). A recent meta-analysis supports a decrease in GABA concentrations in the cingulate gyrus in first-episode psychosis (Nakahara et al., 2022). Despite variability across studies (Egerton et al., 2017), the meta-analysis results are consistent with the reduction of GABA synthesis suggested by postmortem brain studies. It has also been reported that GABA concentrations in the dorsolateral prefrontal cortex are positively associated with working memory, although these findings were only found in healthy individuals (Yoon et al., 2016).

A genome-wide association study identified 108 loci associated with schizophrenia (Ripke et al., 2014). The list included genes that could be related to GABAergic neurons but not GAD67 or PV. In contrast, smaller genetic studies have shown that single nucleotide polymorphisms (SNPs) and loss-of-function mutations in the *GAD1* gene were involved in GAD67 expression levels and schizophrenia (Addington et al., 2005; Straub et al., 2007; Du et al., 2008; Hyde et al., 2011; Giacopuzzi et al., 2017; Magri et al., 2018). These results suggest that there is no direct *GAD1* gene vulnerability in all cases of schizophrenia, but rather that the reduction in GAD67 occurs because of other biological factors. For example, maternal infection, a risk factor for schizophrenia (Brown and Derkits, 2010), can reduce GAD67 levels (Giovanoli et al., 2015; Volk, 2017). Genetic variation in C4, a component of the complement system in the innate immune system, has been associated with an increased risk of developing schizophrenia (Sekar et al., 2016). The observed decrease in GAD67 expression in mice overexpressing C4 protein suggests that changes in the neuroimmune system may influence GAD67 expression (Druart et al., 2021). Other upstream factors, such as decreased input to GABAergic neurons (Akbarian and Huang, 2006), and a reduction in Zif268, a transcription factor that regulates GAD67 expression, in postmortem brains of individuals with schizophrenia have also been reported (Kimoto et al., 2014). Even if upstream biological processes vary, the consistent decrease in GAD67 found in postmortem brain studies may represent a common pathophysiology of the syndrome.

## 3. GAD67 knockout/knockdown animal models

Given that the aforementioned studies were observational in nature, it can be challenging to definitively determine the psychiatric symptoms resulting from decreased GAD67 or GABA levels. However, this can be achieved through the creation of animal models in which GABA synthesis has been experimentally reduced, and its behavior has been examined. This section discusses the phenotypes of rodents with diminished GAD67 expression (Table 1).

**TABLE 1:** Behavioral phenotype comparison of *Gad1* (GAD67) knockout/knockdown animals.

Model animal	Characteristics	References	GAD67 expression level	Body weight	Mortality rate	Open field test	Sensitivity to NMDA receptor antagonist (acute administration)	Elevated-plus maze test or 0-maze test	Light-dark transition test	Y-maze test (working memory)	Eight-arm radial maze test (working memory)
GAD67^–/–^ mice	Homozygous knockout	Asada et al., 1997	Theoretically 0% of WT	normal	**100%**	N.A.	N.A.	N.A.	N.A.	N.A.	N.A.
*GAD67*^+/GFP^ (GAD67^–/–^) mice	Heterozygous knockout	Uchida et al., 2011, 2014; Sandhu et al., 2014; Nullmeier et al., 2020	Theoretically 50% of WT	Normal	–	Normal	N.A.	Normal; **isolation stress induced hyperactivity**	Normal	N.A.	N.A.
LV-Si GAD67 mice	Amigdala-specific knockdown	Heldt et al., 2012	65% of WT (mRNA, amygdala)	Normal	–	Normal	N.A.	Normal; **decreased sensitivity to diazepam**	N.A.	N.A.	N.A.
*Pvalb/Gad1* Tg mice	PV neurons specific knockdown	Brown et al., 2015	N.A.	Normal	–	normal	**Low dose ketamine: increased rearing** **high dose ketamine: decreased rearing**	Normal (0-maze)	Normal	Normal	N.A.
*PV-Cre;GAD67*^flox/+^** mice	PV neurons (plus a part of SST and CR neurons) specific heterozygous knockout	Fujihara et al., 2015a	79% of WT (protein, cerebral cortex)	Normal	–	Normal	**Increased**	Normal	Normal	Normal	N.A.
PV^*GAD*67–/–^ mice	PV neurons specific knockout	Georgiev et al., 2016	60% of WT (protein, somatosensory cortex)	Normal	–	Normal	N.A.	N.A.	**Increased transition number**	Normal; **hyperactivity**	N.A.
*Ppp1r2-Cre^+/–^;Gad1*^loxP/loxP^** mice	Cortical and hippocampal interneurons (including PV neuron) specific knockout	Kolata et al., 2018	50% of WT (protein, somatosensory cotex)	**Increased**	–	**Transient hyperactivity**	Normal	Normal	**Increased time staying in the light chamber**	Normal	N.A.
Doxycycline-treated *Gad1*^tTA/STOP–tetO^* mice*	Adult-stage knockdown	Miyata et al., 2021	11.6% of WT (protein, frontal cortex)	Normal	**57% after doxycycline treatment**	**Hyperactivity**	N.A.	N.A.	N.A.	N.A.	N.A.
*Gad1*^–/–^ rats	homozygous knockout	Fujihara et al., 2020, 2021a	Theoretically 0% of WT	**Low body weight only during developmental stage**	**66%**	**Hypoactivity**	**Increased**	Normal	N.A.	**Impaired**	**Impaired**; **hyperactivity**
*Gad1^–/–^* rats	Heterozygous knockout	Fujihara et al., 2020, Fujihara et al., 2021b	Theoretically 50% of WT	Normal	–	Normal	N.A.	Normal; **slightly shorten stay time on the center region**	N.A.	Normal	N.A.
**Morris water maze test**	**Acoustic startle response**	**Prepulse inhibition**	**Sociability test**	**Social novelty preference**	**Forced swim test**	**Tail-susupension test**	**Fear memory acquisition**	**Fear extinction**	**Intruder aggression test**	**Circadian activity**	**Others**
N.A.	N.A.	N.A.	N.A.	N.A.	N.A.	N.A.	N.A.	N.A.	N.A.	N.A.	Cleft palate, decrease in GABA ∼7% of wild-type.
N.A.	Normal	Normal	**Impaired**	N.A.	**Increased immobility**	N.A.	N.A.	N.A.	**Reduced aggressive behavior**	**2 h delay shift**	Increase in the number of tyrosine hydroxylase (+) fiber in CA1, vulnerable to maternal stress.
N.A.	Normal	N.A.	N.A.	N.A.	N.A.	N.A.	Normal	**Impaired**	N.A.	N.A.	–
N.A.	N.A.	**Impaired**	N.A.	**Increased social novelty preference**	N.A.	N.A.	Normal	**Impaired**	N.A.	N.A.	Unaltered PV neuron density
N.A.	Normal	**Impaired**	Normal	**Impaired**	Normal	N.A.	Normal	Normal	N.A.	N.A.	Upregulation of GAD65 and PV, increased spine density in CA1
N.A.	N.A.	Normal	N.A.	N.A.	N.A.	N.A.	N.A.	N.A.	N.A.	N.A.	Upregulation of GAD65, PV,KCNS3, BDNF and TrkB
N.A.	Normal	Normal	**Impaired**	N.A.	**Increased immobility**	**Increased immobility**	N.A.	N.A.	N.A.	N.A.	Upregulation of GAD65, deficit in dopamine release response to NMDA receptor antagonist (MK-801, 0.2 mg/kg)
N.A.	**Reduced**	Normal	N.A.	N.A.	N.A.	N.A.	N.A.	N.A.	N.A.	N.A.	–
**Impaired**	Normal	Normal	Normal	**Impaired**	**Decreased immobility**	N.A.	**Cued: impaired** **context: normal**	**Context: impaired**	N.A.	N.A.	Upregulation of GAD65 decrease in GABA concentration (∼50% of wild-type)
N.A.	**Reduced**	Normal	Normal	Normal	**Increased immobility**	N.A.	Normal	**Impaired**	N.A.	N.A.	Decrease in GABA/glu ratio

N.A. stands for not assessed. Bold values indicated the behavioral tests that showed significant changes.

### 3.1. GAD67 knockout mice (*Gad1^–/–^* mice)

The ablation of GAD67 in mice (GAD67 knockout mice, also known as *Gad1^–/–^* mice) resulted in a reduction in GABA levels to 7% of that observed in wild-type mice. As embryonic GAD67 serves as the primary GABA-producing enzyme in mice, its complete loss is detrimental to survival, resulting in the manifestation of cleft palate and death shortly after birth (Asada et al., 1997; Kakizaki et al., 2015). Consequently, behavioral analysis of adult animals is infeasible, rendering GAD67 knockout mice unsuitable as a model for investigating psychiatric disorders.

In contrast, GAD67 heterozygous knockout mice (*Gad1^–/–^* mice) survive to adulthood. Female mutants demonstrate increased vulnerability to stress during pregnancy, which leads to reduced body weight in their offspring (Uchida et al., 2011). Furthermore, GAD67 heterozygote fetuses exhibit elevated corticosterone levels and lower body weight than wild-type littermates. Examination of the offspring after birth revealed that the number of PV neurons in the prefrontal cortex was reduced in GAD67 heterozygous knockout mice subjected to maternal stress (Uchida et al., 2014). Regrettably, behavioral experiments have yet to be performed on these mice, thus the presence or absence of behavior analogous to schizophrenia remains unknown. GAD67 heterozygous knockout mice exhibit decreased sociability and aggressiveness in the absence of maternal stress (Sandhu et al., 2014), as well as elevated immobility during the forced swim test (Nullmeier et al., 2020). Furthermore, social isolation stress elicits an increase in locomotor activity in GAD67 heterozygous knockout mice, but does not elicit a similar response in their wild-type littermates. Impaired social behavior and stress-induced hyperactivity may reflect negative and positive symptom-like behaviors, respectively. Histological analysis revealed that tyrosine hydroxylase-positive nerve fibers in the hippocampal CA1 region were increased, suggesting dopaminergic neural changes even under maternal stress-free conditions (Nullmeier et al., 2020). In summary, while the phenotype of *Gad1^–/–^* mice exhibits some similarities to schizophrenia, the effect it has on working memory remained inconclusive.

### 3.2. Conditional GAD67 (*Gad1*) knockout/knockdown mice

As noted earlier, the postmortem brains of patients with schizophrenia show a significant decrease in GAD67 expression, especially in PV neurons. Several groups have created conditional knockout mice that mimic this finding. Two of these mouse models (*Pvalb/Gad1*-silencing mice and *PV-Cre; GAD67^+/flox^* mice) showed altered prepulse inhibition and responsiveness to NMDA receptor antagonists and a partially schizophrenia-like phenotype but no apparent working memory impairment (Brown et al., 2015; Fujihara et al., 2015a). However, because both mouse models were evaluated only with the Y-maze task, a very simple spatial working memory task, more sensitive tasks such as the eight-arm radial maze test will be needed (Olton and Samuelson, 1976; David and Olton, 1979). Another strain of PV neuron-specific GAD67 knockout mice (PV^*GAD*67^*^–/–^* mice), reported by Georgiev et al. (2016), showed a phenotype different from the first two, as they did not show impaired prepulse inhibition. Moreover, their locomotor activity increased in the light/dark transition and Y-maze tests without affecting working memory. They analyzed the cerebral cortex of mice using a method similar to that used in postmortem brain studies (*in situ* hybridization). They found increased expression of PV, GAD65, brain-derived neurotrophic factor (BDNF), and TrkB, a profile that differs from the actual postmortem findings in schizophrenia. Among these, the increased expression of PV and GAD65 was consistent with the mouse model of Fujihara et al. (2015a). As well-known, the BDNF-TrkB signaling pathway was attenuated in schizophrenia. From this, they speculated that the reduced expression of GAD67 in PV neurons may be downstream of the disease pathway and not enough to produce schizophrenia-like behaviors and histological characteristics (Georgiev et al., 2016). Another study generated a conditional knockout mouse that covers more widespread GABAergic neurons, including PV neurons (*Ppp1r2-Cre^+/–^; Gad1*^loxP/loxP^**; Kolata et al., 2018). This mouse model is inconsistent in its behavioral phenotype with the first two conditional knockout mice, as it shows no impairment in prepulse inhibition and does not respond to NMDA receptor antagonist administration. However, it displayed increased mobility in the forced swim test and reduced sociability in the social interaction test, showing similarity to GAD67 conventional heterozygous knockout mice. In addition, this mouse model had no tail-suspension-induced dopamine release in the anterior cingulate cortex. It has been noted that these findings may be related to negative symptoms of schizophrenia (Kolata et al., 2018).

### 3.3. Site-specific and inducible GAD67 (*Gad1*) knockdown mice

Site-specific GAD67 knockdown mice also provided insight into the association between GAD67 and psychiatric symptoms. A lentiviral RNA interference strategy was used to perform amygdala-specific knockdown of GAD67 (Heldt et al., 2012). The model mice displayed impaired fear memory elimination, as in *Pvalb/Gad1*-silencing mice (Table 1). Time-specific GAD67 global knockdown mice have also been developed. *Gad1^tTA/STOP–tetO^* mice show a progressive loss of GAD67 expression upon doxycycline treatment at the adult stage (Miyata et al., 2021). This process causes mice to exhibit hyperlocomotion and anxiety-like behaviors. These results suggest that a decrease in GAD67 also results in emotional abnormalities.

### 3.4. GAD67 (*Gad1*) knockout rats

Although the model animals described above are mice, recently, GAD67 knockout rats (*Gad1^–/–^* rats) have been generated using the CRISPR/Cas9 system. Unlike *Gad1^–/–^* mice, they do not exhibit cleft palate, and approximately 33% of individuals can grow into adults (Fujihara et al., 2020; Jiang et al., 2022). The mortality rate of *Gad1^–/–^* rats after adulthood is the same as that of wild-type rats, allowing for long-term breeding and behavioral tests. *Gad1^–/–^* rats show reduced spontaneous locomotion, hypersensitivity to NMDA receptor antagonists, and impaired social recognition memory, spatial reference memory, spatial working memory, and cued-fear memory (Fujihara et al., 2020, Fujihara et al., 2021b). These symptoms may correspond to negative symptoms, positive symptoms, and cognitive dysfunction. This rat model is the only one that exhibits a distinct working memory deficit among GAD67 knockout/knockdown animals. In contrast, *Gad1^–/–^* rats showed only very mild behavioral changes that did not necessarily match the phenotype of *Gad1^–/–^* mice (Fujihara et al., 2021a). The authors repeatedly exposed *Gad1^–/–^* rats to a low-dose NMDA receptor antagonist during adolescence but did not find as many behavioral abnormalities as *Gad1^–/–^*. Of note, *Gad1^–/–^* rats also showed an increase in GAD65 levels in the cerebral cortex, similar to the conditional knockout mice (Fujihara et al., 2020). Although this compensatory increase is not seen in schizophrenia, a higher level of GAD65 is not sufficient to prevent behavioral alterations in *Gad1^–/–^* rats (Figure 1C).

### 3.5. Comments on GAD67 (*Gad1*) knockout/knockdown animals

One major limitation in model animal research is the species difference between humans and rodents. For example, it has been established that humans have specialized GABAergic neurons (Boldog et al., 2018) and different GABAergic neuron markers compared to rodents (Krienen et al., 2020). Although it is unclear how these differences affect behavior, they may contribute to phenotypic differences between human schizophrenia and animal models. In addition, it should be noted that even when the same gene is knocked out, phenotypic differences may exist between mice and rats (Fujihara et al., 2020), highlighting the importance of considering species differences in future research.

Glutamic acid decarboxylase 67 knockout animals show a variety of behavioral changes, some of which may be related to schizophrenia. However, the symptom domains that are affected depend on the animal species, the method used, and the extent of knockout/knockdown. Additionally, certain model animals exhibit phenotypes that are diametrically opposed to one another (e.g., locomotor activity in the open field test; see Table 1). Patients who have schizophrenia may manifest psychomotor agitation in conjunction with positive symptoms or alternatively, they may display a diminished level of activity with negative symptoms predominating. If reduced GAD67 is involved in both positive and negative symptoms in human schizophrenia, it is unclear what would cause such variability. It may be possible to answer this question by analyzing what makes animals with similar genetic vulnerabilities different. For example, the difference between *Gad1^–/–^* rats and doxycycline-treated *Gad1^tTA/STOP–tetO^* mice is that, in addition to species differences, GAD67 is deficient from the embryo in the former, whereas in the latter, the decrease proceeds as an adult (Fujihara et al., 2020; Miyata et al., 2021). Nevertheless, no clear rule of thumb on the phenotype differences among GAD67 knockout/knockdown animals can be found at this time.

The impairment in fear extinction is the most commonly observed phenotype in the GAD67 knockout/knockdown model animals (Table 1). Individuals with schizophrenia also have impairments in extinction memory recall, which is associated with decreased ventromedial prefrontal activity in patients (Holt et al., 2009, 2012). In schizophrenia, the functional connectivity between amygdala and medial prefrontal cortex is diminished. The amygdala-specific GAD67 knockdown is sufficient to cause this phenotype, suggesting GABAergic dysfunction in amygdala underlies the abnormal fear memory in schizophrenia.

Notably, although working memory deficits are thought to be derived from abnormalities in the GABAergic nervous system, they have only been observed in *Gad1^–/–^* rats (Fujihara et al., 2020). Theoretically, the capacity for GABA production in *Gad1^–/–^* rats is the lowest among the model animals that can undergo behavioral tests. As the reduction of GAD67 is supposed to be milder in human schizophrenia, we can speculate that another biological factor (s) possibly interacts with GAD67 reduction, resulting in working memory impairment.

## 4. Interaction between GABAergic system and neuroimmune system

Finally, we discuss a perspective that has not yet been evaluated using these animal models. Psychological stress, which is crucial for the development of psychiatric disorders (Norman and Malla, 1993), can induce immune activation (Volk, 2017). If the neuroimmune system is altered by GABAergic dysfunction in schizophrenia, it may also affect the progression of the disorder. Although GABA-A receptor expression in microglia is unknown (Barragan et al., 2015), microglia express GABA-B receptors, which alter K^+^ permeability upon GABA signaling. Microglia release interleukin-6 (IL-6) in response to stimulation with lipopolysaccharides, but this release is inhibited by the simultaneous activation of GABA-B receptors (Kuhn et al., 2004). Taken together, it is also speculated that microglia are more likely to be activated under low GABA conditions, such as schizophrenia.

Yeung et al. (2018) have already reported the relationship between the GABAergic system and microglia in a model animal of schizophrenia. Mice with knockout of the β2 subunit of the GABA-A receptor (*Gabrb2* knockout mice) show an increased number of microglia in the cortex and hippocampus (Yeung et al., 2018), accompanied by increased levels of cytokines such as TNF-α and IL-6. In schizophrenia patients, the serum IL-6 level is also elevated in untreated schizophrenia by approximately 1.5 standard deviation above that in healthy controls (Zhou et al., 2021). At present, there is no evidence that GABA-A receptors, which contain the β2 subunit, exist in microglia. Thus, the mechanism of microglial activation in *Gabrb2* knockout mice is different from that discussed in the previous paragraph. Yeung et al. (2018) speculated that microglial activation is caused by an astrocyte-mediated mechanism in *Gabrb2* knockout mice.

Recently, microglia with GABA-B receptors have been found to be involved in pruning GABAergic synapses (Favuzzi et al., 2021). Loss of microglia-specific GABA-B receptors significantly impairs GABAergic synaptic pruning. As a result, the number of GABAergic synapses increased at P30 but decreased by P60. The mouse model was hypoactive at P30 and hyperactive at P60. Although Favuzzi et al. (2021) noted an association with attention deficit hyperactivity disorder (ADHD), the transition from hypoactivity to hyperactivity is not identical to the course of ADHD in humans. However, the change in symptoms as the animal reaches adulthood and the marked reduction in the number of GABAergic terminals also reminds us of an association with schizophrenia.

Collectively, these findings suggest that the disruption of inhibitory neurotransmission may create an environment in which microglia are more likely to become activated, further altering the pathology via microglial responses. In *Gad1^–/–^* rats, tissue GABA levels were reduced to approximately 50% of those in wild-type rats (Fujihara et al., 2020). Although this reduction may be too extreme compared with schizophrenia patients, *Gad1^–/–^* rats are expected to be a good experimental system to investigate the extent to which reduced tissue GABA levels affect microglial properties (Figure 1C). Imposing stress or more direct inflammation in these systems may also be useful in elucidating the mechanisms by which environmental factors and predisposing factors interact to cause disease progression.

## 5. Conclusion

There is no guarantee that schizophrenia is a single biological disorder; rather, it is regarded as a syndrome conceptualized based on psychopathological symptoms and course. Therefore, biological studies on schizophrenia should focus on “common pathways” shared by as many patients as possible and on therapeutic interventions to address them rather than on a complete understanding of all cases.

Just as conventional therapies targeting the dopaminergic nervous system are not perfect, it may be too much to hope for the ideal efficacy of new treatment strategies. However, in schizophrenia research, where major breakthroughs have not been achieved since the discovery of antipsychotics, it is expected that as many therapeutic strategies as possible will be added to this mix. The disturbance in GABAergic system remains a promising candidate as a therapeutic target, because of its replicability in the postmortem brain studies.

To produce significant working memory deficits in rodents, complete loss of GAD67 was required. This is a considerably more severe abnormality than that observed in postmortem brain studies. Therefore, in many actual cases of schizophrenia, a milder abnormality in the GABAergic nervous system may combine with other biological factors to form working memory impairment. This could be an abnormality of excitatory input onto GABAergic neurons (Figure 1B), or it could be an interaction between specific environmental factors and GABAergic abnormalities. To test these possibilities, it would be helpful to expose the GAD67 mutants discussed here to various environmental factors, including stress. To date, there have been no reports of stress-induced cognitive dysfunction in GAD67 mutants. However, it can cause activity changes in *Gad1^–/–^* rats (Nullmeier et al., 2020). Analysis of the environmental factors that interact with the vulnerability of GABAergic neurons, the timing and mechanism of these interactions, and the resulting phenotypic changes can lead to a better understanding of schizophrenia’s pathophysiology. In doing so, it will be necessary to pay attention to the intervention of microglia and other factors, rather than focusing only on neurons, as has been done previously.

## Author contributions

KF reviewed the literature, wrote the manuscript, and approved the submitted version.
